# Evolução Temporal da Análise de Resultados do Emprego do iFR

**DOI:** 10.36660/abc.20200195

**Published:** 2020-10-13

**Authors:** Maria Cristina Meira Ferreira, Gláucia Maria Moraes de Oliveira

**Affiliations:** 1 Hospital Federal dos Servidores do Estado Rio de JaneiroRJ Brasil Hospital Federal dos Servidores do Estado - Serviço de Hemodinâmica, Rio de Janeiro, RJ - Brasil; 2 Universidade Federal do Rio de Janeiro Rio de JaneiroRJ Brasil Universidade Federal do Rio de Janeiro, Rio de Janeiro, RJ – Brasil

**Keywords:** Isquemia Miocárdica, Reserva Fracionada do Fluxo do Miocárdio, Stents, Doença da Artéria Coronariana, Intervenção Coronária Percutânea/métodos

Senhor Editor,

Lemos com muito interesse o minieditorial escrito pelos autores Chamié e Abzaid^1^ referente ao artigo “Avaliação de isquemia miocárdica na sala de hemodinâmica com *instanataneous wave-free ratio*: estudo piloto”.^2^ O minieditorial nos traduz com clareza a histórica evolução de raciocínio que devemos seguir na interpretação do estudo de fisiologia coronária na tomada de decisão terapêutica. Embora a medicina seja plena de situações binárias para resolução, como presença ou ausência de febre pelo termômetro, fica muito claro que níveis diferentes de valores referem-se a diagnósticos, prognósticos e condutas diferentes. Com relação às avaliações funcionais coronarianas, após uma enorme quantidade de estudos binários para demonstrar sua validade, recentes *trials* citados no minieditorial nos conduzem a uma fase onde o poder decisório clínico volta a ter peso importante e,^3^ o dissertar desta mudança de rumos ocorreu de forma brilhante pelo minieditorial. Não deixamos de relevar o raciocínio clínico e outros fatores em nosso estudo, uma vez que o preditor de colocação de *stent* foi iFR < 0,87 neste grupo, apesar do valor de corte estabelecido para o iFR ser 0,89, havendo uma significativa redução do emprego de *stents*.

Cabe-nos ressaltar que nosso estudo foi realizado com dados coletados no período de 2014 a 2018, abrangendo um longo período em que o iFR ainda não tinha o corte binário bem estabelecido. Até a publicação dos grandes *trials Swedeheart e Define-Flair* em 2017, considerava-se os valores de iFR > 0,86 e iFR<0,93 como zona cinzenta, e as diretrizes referentes ao método indicavam emprego do *fractional flow reserve* (FFR).^4^^,^^5^ Neste cenário de tempo, a colocação de *stents* em pacientes com valores de iFR ≤ 0,92 não pode ser considerada desnecessária como citada, pois havia carência de dados literários que corroborassem de forma definitiva o corte de 0,89, que só ocorreu após comparação entre os métodos FFR e iFR nos *trials* citados.

Agradeço a oportunidade de poder esclarecer estes pontos e corroboro que o minieditorial nos direciona e nos esclarece não só a necessidade de cada vez mais utilizarmos a fisiologia coronária, mas também de como utilizá-la nos dias de hoje, contribuindo de forma primorosa neste campo de estudo da cardiologia intervencionista.
