# Supplementary material for: Evolução Temporal da Análise de Resultados do Emprego do iFR
Source: Arq Bras Cardiol. 2020 Oct 13;115(4):719. [Article in Portuguese] doi: 10.36660/abc.20200195 (PMC8386980; doi:10.36660/abc.20200195)
Supplement: Translation

# Translation: Temporal Evolution of the iFR (Instantaneous Wave-Free Ratio) Employment Results Analysis

**Authors:** Maria Cristina Meira Ferreira, Gláucia Maria Moraes de Oliveira

**Affiliations:** 1 Rio de JaneiroRJ Brazil Hospital Federal dos Servidores do Estado - Serviço de Hemodinâmica, Rio de Janeiro, RJ - Brazil; 2 Rio de JaneiroRJ Brazil Universidade Federal do Rio de Janeiro, Rio de Janeiro, RJ – Brazil

**Keywords:** Myocardial Ischemia, Fractional Flow Reserve, Myocardial, Stents, Coronary Artery Disease, Percutaneous Coronary Intervention/methods

Dear Editor,

We read with interest the short editorial written by authors Chamié and Abzaid^1^ regarding the paper “Evaluation of Myocardial Ischemia with iFR (instantaneous wave-free ratio) in the catheterization laboratory: a pilot study”.^2^ The short editorial clearly translates to us the historical evolution reasoning that we must follow when interpreting coronary physiology studies in therapeutic decision-making. Although medicine is full of binary situations for resolution, such as the presence or absence of fever by the thermometer, it is very clear that different levels of values refer to different diagnoses, prognosis and treatments. With regard to coronary functional assessments, after an enormous amount of binary studies to demonstrate their validity, recent trials cited in the short editorial directed us to a phase where clinical decision-making power has an important weight once again,^3^ and the dissertation of this change in direction occurred brilliantly in the editorial. We did not neglect the clinical reasoning and other factors in our study, since the stent placement predictor was iFR < 0.87 in this group, despite the cutoff value established for iFR being 0.89, with a significant reduction in the use of stent.

It is worth mentioning that our study was conducted with data collected from 2014 to 2018, covering a long period in which the iFR did not have a still well-established binary cutoff value. Until the publication of Swedeheart and Define-Flair trials in 2017, the values of iFR > 0.86 and iFR < 0.93 were considered as gray zone, where the guidelines for the method indicated the use of *fractional flow reserve* (FFR).^4^^,^^5^ In this time scenario, the placement of stents in patients with value of iFR ≤ 0.92 cannot be considered unnecessary as mentioned, due to the lack of literary data that definitively corroborated the 0.89 cutoff value, which only occurred after comparison between the FFR and iFR methods in the trials aforementioned.

I am grateful for the opportunity to clarify these points and corroborate that the editorial directs us and clarifies not only the need to increasingly use coronary physiology, but also how to use it today, contributing exquisitely in this area of interventional cardiology study.
